# Color and Stability of Anthocyanins of Chagalapoli (*Ardisia compressa* K.) Fruit Added to an Isotonic Beverage as Microcapsules and as Free Extract

**DOI:** 10.3390/foods12102009

**Published:** 2023-05-16

**Authors:** María Vianey Antonio-Gómez, Yolanda Salinas-Moreno, Francisco Hernández-Rosas, José Andrés Herrera-Corredor, Adriana Contreras-Oliva

**Affiliations:** 1 Colegio de Postgraduados, Campus Córdoba, Postgrado Innovación Agroalimentaria Sustentable, Km 348 Carretera Córdoba-Veracruz, Amatlán de los Reyes, Veracruz 94946, Mexico; 2 Instituto Nacional de Investigaciones Forestales, Agrícolas y Pecuarias, Campo Experimental Centro Altos de Jalisco, Km 8 Carretera Tepatitlán-Lagos de Moreno, Tepatitlán de Morelos 47600, Mexico

**Keywords:** *Ardisia compressa* K., anthocyanins, microcapsules, isotonic beverages, color

## Abstract

The demand for natural pigments in the food industry is increasing. Color and stability of anthocyanins of chagalapoli (*Ardisia compressa* K.) fruit added to an isotonic beverage as microcapsules and free extract were evaluated at two temperatures (4 and 25 °C) in the absence of light. Anthocyanins degradation followed first-order kinetics in the evaluated conditions. The stability of anthocyanins, measured by the variables reaction rate (K), half-life time (t_1/2_), and anthocyanin retention (AR), was affected significantly (*p* < 0.01) by temperature. At the end of storage at 4 °C, AR was 91.2 ± 0.28% and 89.63 ± 0.22% in the beverages with microcapsules (BM) and with anthocyanins from extract (BE), respectively, without a significant difference (*p* ≥ 0.05) between them. However, at 25 °C, AR in the BM was 53.72 ± 0.27%, a significantly lower value (*p* ≤ 0.05) than that in BE (58.83 ± 1.37%). The color difference values (ΔE) in beverages stored at 4 °C were 3.81 and 2.17 for BM and BE, respectively, while at 25 °C, it was 8.57 and 8.21, respectively. The most stable anthocyanin was cyanidin 3-galactoside. Chagalapoli anthocyanins, both as microcapsules or as an extract, are adequate for adding natural color to isotonic beverages.

## 1. Introduction

Color is one of the most important properties of foods and a factor that definitively influences consumers at the time of selecting food [1]. Color is added to processed foods to restore color that is lost during processing, to increase existing color or simply to add color to colorless foods or beverages [2]. The substitution of several synthetic colorants for natural pigments has increased substantially in the European Union [3]. The increase has been attributed to legislative changes in several countries based on research results that link synthetic colorants with health problems, such as allergies [4], and hyperactivity in children [5], and their possible relationship with chronic degenerative diseases. Another reason that pushes the food industry to use natural pigments rather than synthetic ones is the consumers demand for all natural foods, which they perceive as healthier. This situation has stimulated the market for natural pigments, which currently is increasing [6].

Anthocyanins are considered potential substitutes for synthetic colorants, particularly those with red tones, due to the brilliant and attractive colors they impart to foods [7]. Anthocyanins are flavonoid compounds, innocuous and easily incorporated in aqueous media because of their high solubility [8]. In addition to adding color, anthocyanins posse high antioxidant capacities, which makes them valuable in preventing the development of chronic degenerative diseases [9]. However, their stability in foods can be affected by several factors, such as pH, storage temperature, light, and oxygen, among others [10].

The spray-drying microencapsulation technique is widely used in the food industry to improve the stability of various natural compounds susceptible to degradation by external agents and to facilitate its manipulation during food formulation. It has been successfully applied to microencapsulate anthocyanins from various plant sources to improve their stability [11] and facilitate their use because some dried extracts of anthocyanins present a sticky consistency that makes difficult its manipulation [12].

*Ardisia compressa* K. is a wild bush-tree growing in the tropical and subtropical areas of Mexico. Its fruit, commonly named chagalapoli or acachul, depending on the country region, is a drupe with a high content of anthocyanins (7.96 g/kg of fresh fruit), and a profile in which malvidin, delphinidin, and petunidin galactosides dominate [13]. This fruit is a potential source for the extraction of anthocyanins, which for their particular composition can impart tones of color different from those obtained with anthocyanins based in cyanidin derivatives [14]. The optimized process of extracting the pigments from this fruit and their microencapsulation with mixtures of maltodextrin and Capsul^®^ as wall material has been recently reported [15]. However, the application of microencapsulated anthocyanins to add color to foods and beverages has not been studied. Therefore, in the present study, the stability of the color and anthocyanins of the chagalapoli fruit (*Ardisia compressa* K.) was evaluated; both in microcapsules and in free extract, in an isotonic drink stored under two temperature conditions.

## 2. Materials and Methods

### 2.1. Reagents and Plant Material

Chemical substances used included analytical reagent-grade ethanol, hydrochloric acid, formic acid, and methanol (J.T.Baker^®^ VWR, Tlanepantla, Mexico). High-performance liquid chromatography (HPLC)-grade water and methanol were used for anthocyanin analysis. Commercial standards of malvidin 3-O-galactoside and delphinidin 3-O-galactoside were purchased from Extrasynthese (Genay, France), while cyanidin 3-O-glucoside, malvidin 3- O-glucoside, and delphinidin 3-O-glucoside were purchased from polyphenols (Sandnes, Norway). The wall materials used were Capsul^®^ (National Starch, Argo, IL, USA) and maltodextrin 10 DE (IMSA, Guadalajara, Jalisco. Mexico).

The plant material consisted of mature fruits from chagalapoli (*A. compressa* K.), which were obtained from the regional market of San Andrés Tuxtla, Veracruz. The pit of the fruit was manually removed, and the pulp was homogenized with an Ultra-Turrax^®^ homogenizer (Basic T-10, IKA, San Diego, CA, USA) for 1 min at 20,450 rpm.

### 2.2. Obtaining Anthocyanins Extract

For obtaining the anthocyanin extract, we follow the protocol described by [15], in which the study optimized the anthocyanins extraction from chagalapoli fruit. Homogenized fruit pulp was mixed in a 1:5 proportion (sample: solvent) with 65% aqueous ethanol. The pH was adjusted to 2.5 with hydrochloric acid, and the mixture was placed in an ultrasonic bath (Model 2510, Branson,Hampton, NH, USA) for 30 min and then subjected to agitation for 30 min in a horizontal agitator at room temperature (~25 °C) protected from light. The extract was recovered by centrifuging (Universal 32 Model centrifuge, Hettich, Buford, GA, USA) the sample at 2200× *g* for 10 min. Finally, to eliminate ethanol from the system, the extract was concentrated in a rotary evaporator (R-215, Buchi, Rose Scientific Ltd., Edmonton, AL, Canada) at 40 °C until reduced to one-third of the initial volume. The concentrated extract contained 94.90% ± 1.27 moisture, 11.4 ± 0.02° Brix, 5.10% ± 1.27 total solids, and a pH of 2.72 ± 0.02.

### 2.3. Preparation of Spray-Dried Microcapsules

The suspension was prepared at a final concentration of 20% (*w*/*v*). A total of 50 g of wall material (25% 10DE maltodextrin and 75% Capsul ^®^) was used and dissolved with 200 mL of distilled water in a household blender (Waring) for 1 min at low speed. The rate of maltodextrin:Capsul used was reported by [15] as the best for anthocyanin microcapsules stability. Subsequently, the mixture was homogenized in an Ultra-Turrax device (Ultra-Turrax T-25-SI, IKA Works, Wilmington, NC, USA) at 18,000 rpm for 5 min. A total of 50 mL of concentrated anthocyanin extract was added, and homogenization was continued for 10 additional minutes. Encapsulation was performed using a spray dryer (SD-Basic Lab-Plant, Huddersfield, UK) under the following drying conditions: air inlet temperature 160 ± 1 °C, air outlet temperature 95 ± 5 °C, 35 Psi pressure 0.5 mm nozzle diameter, and a feed stream of 10 mL/min [15]. The powders were collected in plastic bags, weighed, and stored under refrigeration in a silica desiccator. The powdered pigment had 2.74 ± 0.40% moisture and 0.11 ± 0.00 water activity. Its total anthocyanin content (TAC) was 10.74 ± 0.24 mg/g of powder.

### 2.4. Preparation of the Isotonic Beverage and Addition of Color from Chagalapoli Anthocyanins in Microcapsules (AM) and in Extract (AE)

Beverage preparation was performed according to the formulation used by [16], which incorporates sucrose (7.5 g), sodium chloride (20 mg), potassium phosphate (6 mg), potassium sorbate (33 mg), and citric acid (300 mg) to obtain a pH of 2.5 per 100 mL of beverage. The pH of the beverage was measured with a Beckman Coulter^®^ Series 45 potentiometer (Beckman Coulter^®^, Inc., Brea, CA, USA). To determine the quantity of anthocyanins to add to the beverage, the color of a commercial beverage (Gatorade^®^, fruit punch flavor) was adopted as a color reference. Tests were performed to determine the quantity of anthocyanins to add (AM or AE). According to the TAC of the MC, it was required around 0.65 g of MC for 100 mL of beverage. For AE, it was used a very concentrated extract from which was needed. In each case, the color was determined using the Hunter-Lab equipment. The beverages were stored in 50 mL glass tubes (a volume of 30 mL of beverage was added each), which were covered and protected from light with aluminum foil. The tubes of beverage were stored at 4 ± 1 °C in a household refrigerator (Whirlpool, Benton Port, MI, USA) and an incubator at 25 ± 1 °C (Binder^®^ Gmbh, Tuttlingen, Germany). The evaluated treatments were as follows: (1) beverage with added anthocyanin extract (AE), stored at 4 °C (BE4) and 25 °C (BE25) and (2) beverage with added microencapsulated anthocyanins (AM) stored at 4 °C (BM4) and 25 °C (BM25). The experiments were performed in triplicate for each temperature.

### 2.5. Analyses Performed on the Beverages at the Beginning and during Storage

On each sampling date, samples were filtered with Whatman No. 4 paper and subsequently analyzed for color, total soluble solids (TSS) (° Brix), pH, and total anthocyanin content (TAC). These variables were determined at the moment of preparing the beverage (time 0) and subsequently weekly for 42 days of storage. At each sampling, three replicates were analyzed per beverage type. Each replicate consisted of a tube that contained 30 mL of beverage.

Color: Determination of this variable was performed as suggested for transparent liquids. The sample was placed in a transparent glass container (Accessory 04-7209-00, HunterLab, Hunter Associates Laboratory, Inc., Reston, VA, USA) and covered with an opaque black metallic cover (Accessory 04-4000-00, HunterLab) to prevent light diffusion. The L* a* b* parameters were obtained in CIELAB scale with a HunterLab^®^ colorimeter (MiniScan Model, Hunter Associates Laboratory, Inc., Reston, VA, USA). Color difference values (Δ*E*) were calculated to determine the changes in color in the beverage over time. Δ*E* was defined as follows: $\Delta E={\left[{\left(L_{i}-L_{0}\right)}^{2}+{\left(a_{i}-a_{0}\right)}^{2}+{\left(b_{i}-b_{0}\right)}^{2}\right]}^{0.5}$, where *L*_0_*, a*_0_ and *b*_0_ are values of the sample at time zero and *L_i_, a_i_*, and *b_i_* are values measured over time [17].

Soluble solids and pH: TSS was measured with a refractometer (PAL-3^®^ model, Atago Co. Ltd., Tokyo, Japan) and expressed in °Brix. The pH was determined with a Beckman Coulter^®^ Series 45 potentiometer (Beckman Coulter^®^, Inc., Brea, CA, USA).

Total anthocyanins content (TAC). This variable was determined with a spectrophotometric method [18] with some adaptations [19]. Briefly, an aliquot of beverage in each sampling date was diluted with acidic water (pH = 2.5), and the absorbance was measured at 530 nm. TAC was expressed as a function of malvidin 3-O-galactoside (Extrasynthese, Genay, France), with which a standard curve was developed. The results are reported in µg equivalents of Mv3gal (MGE)/mL beverage.

The beverage TAC data for each sampling date were processed [20], whereby the natural logarithm of total anthocyanins content was graphed as a function of time for each treatment. The first-order kinetic constant (*k*) and the half-life time (*t*_½_) of the anthocyanins contained in the beverage were obtained using Equations (1) and (2).

(1) Slope of the line=−k/2.303

(2) $$t_{1/2}=-\ln0.5\;x\;k^{-1}$$

The retention percentage of anthocyanins was calculated with Equation (3) [20], where *A_t_* and *A*_0_ correspond to absorbance at time t and time zero, respectively.

(3) $$\%AR=\left(\frac{A_{t}}{A_{0}}\right)100$$

### 2.6. HPLC Analysis of Anthocyanins in Stored Beverages

Anthocyanin analysis was performed at the beginning and at the end of storage in addition to analyzing the AM and AE. A Perkin-Elmer^®^ Series 200 HPLC (PerkinElmer^®^ Instruments LLC, Shelton, CT, USA) was used and operated with TotalChrome^®^ software. The analytical column was Hypersil ODS C18 (200 × 4.6 mm) with a particle size of 5 μm (Thermo Scientific^®^, Waltham, MA, USA). For the analysis, the method of [21] was applied, with some adjustments [19], in a system of linear gradients. The following solvents were used: phase A (1:9 *v*/*v*) (formic acid/water) and phase B (1:4:5 *v*/*v*/*v*) (formic acid/water/methanol). The flow rate was 1.2 mL/min, and the injection volume was 10 µL, with a column temperature of 30 °C. The samples were filtered with a 0.20 µm Millex-LG^®^ film before being injected into the equipment. Anthocyanins detection was made at 520 nm with a UV/vis DAD. The identification of the anthocyanins was performed using commercial anthocyanin standards. Malvidin 3-galactoside, delphinidin 3-galactoside, (Extrasynthese, Genay, France), malvidin 3-glucoside, delphinidin 3-glucoside, and cianidin 3-glucoside (Polyphenols, Sandnes, Norway), and the support of previous reports [13]. The quantification of different anthocyanins was performed based on a standard curve of malvidin 3-O-galactoside.

### 2.7. Statistical Analysis

Data were subjected to a one-way ANOVA analysis. However, we have two types of anthocyanins added to beverages (AM and AE), and two storage temperatures, our interest was to evaluate the combination of these factors on variables related to anthocyanin stability rather than the effect of each factor. The comparison of treatment means was achieved using Tukey’s test (*p* < 0.05). Pearson’s correlation analysis between the variables measured in the drink was performed with the SAS^®^ software version 9.1. Linear regression analysis was performed with Excel 2007 to obtain rate constants in the kinetic studies of anthocyanin degradation in isotonic drinks.

## 3. Results and Discussion

### 3.1. Color Stability in Beverages during Storage

**Figure 1:**
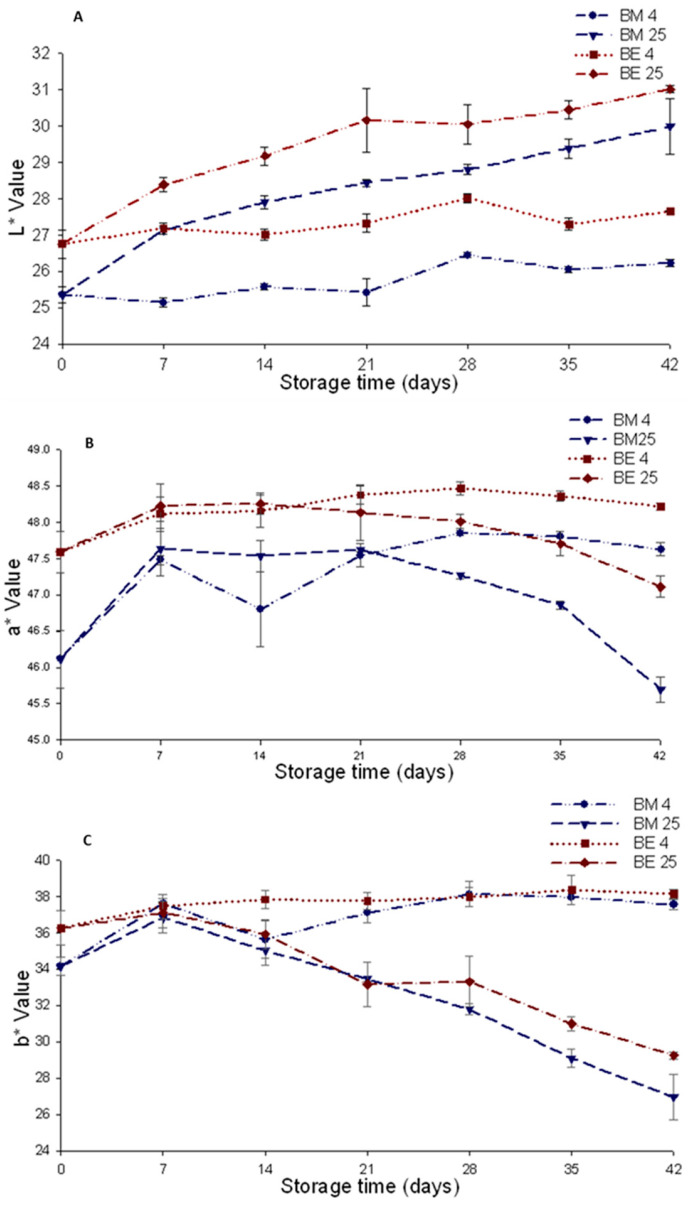
CIEL* a* b* color parameters in isotonic beverages with added chagalapoli anthocyanins as microcapsules or extract stored at 4 and 25 °C. Luminosity (L), BM4: beverage with microcapsules stored at 4 °C. (**A**): L (luminosity), (**B**): a* (+ red, - green), (**C**): b* (+ yellow, - blue). BM25: beverage with microcapsules stored at 25 °C; BE4: beverage with extract at 4 °C; BE25: beverage with extract at 25 °C.

The color of the beverages was expressed in terms of the CIELAB variables L*, a*, and b*. At the start of storage, luminosity (L*) differed between the beverages. The values for this variable were 25.36% and 26.75% for the BM and BE beverages, respectively. The L* value increased with storage; the largest changes were observed in the beverages stored at 25 °C. In the BM beverage, L* increased from 25.36% to 29.99%, while in BE, it increased from 26.76% to 31.02% (Figure 1A). The relative percentage of increase of L* was similar when anthocyanins were added as microcapsules (M) or extract (E). Storage at 4 °C resulted in a minor change of L* in the beverages coloring with AM or AE.

The increase in L* during storage means that the beverages lost color, so they were more luminous. The variable L* presented a high negative correlation (r2 = −0.94616, *p* < 0.0001) with the content of total anthocyanins in the beverages (Table S1), so its increase was associated with the degradation of these pigments. Aguilera et al. [14] observed similar behavior in the L* values in a sports drink stained with bean anthocyanins, and storage for six weeks at temperatures of 4 and 25 °C, although in this case, the L* values were much higher (72–76%), indicative of a less intense color than that of the beverages in the present study.

The behavior of a*, which measures the redness of the beverage, was variable during the storage period. In both beverages, a* value increased within the first seven days of storage. This value remains without greater changes in the period from seven to 21 days. Later, it decreased in the beverages stored at 25 °C (i.e., BM25 and BE25). In the beverages stored at 4 °C, the a* value displayed a new increase, which was more modest than that observed within the first seven days (Figure 1B). The pattern displayed by a* during the beverage storage was not influenced by the condition of the anthocyanins in the beverage (AM or AE); however, a* changes were greater in the beverage with AM. The changes in the a* values indicate that the beverages stored at 25 °C reduced their red tint, while those stored at 4 °C increased it. In this respect, a decrease in a* in a Kool-Aid^®^-based beverage with added purple corn anthocyanins subject to high temperatures (70 to 90 °C) was reported [22]. This behavior is consistent with that observed in the beverages stored at 25 °C in the present study.

The b* value, which measures yellowness, increased slightly within the first seven days of storage. After this time, the beverages stored at 25 °C exhibited a decrease that was associated with storage time, while those stored at 4 °C maintained their value, with a slight increase towards the end of storage (Figure 1C). This pattern was independent of the form in which anthocyanins were added. According to these results, the beverages at 25 °C had a less yellow tone at the end of storage, while those stored at 4 °C were slightly more yellow.

**Figure 2:**
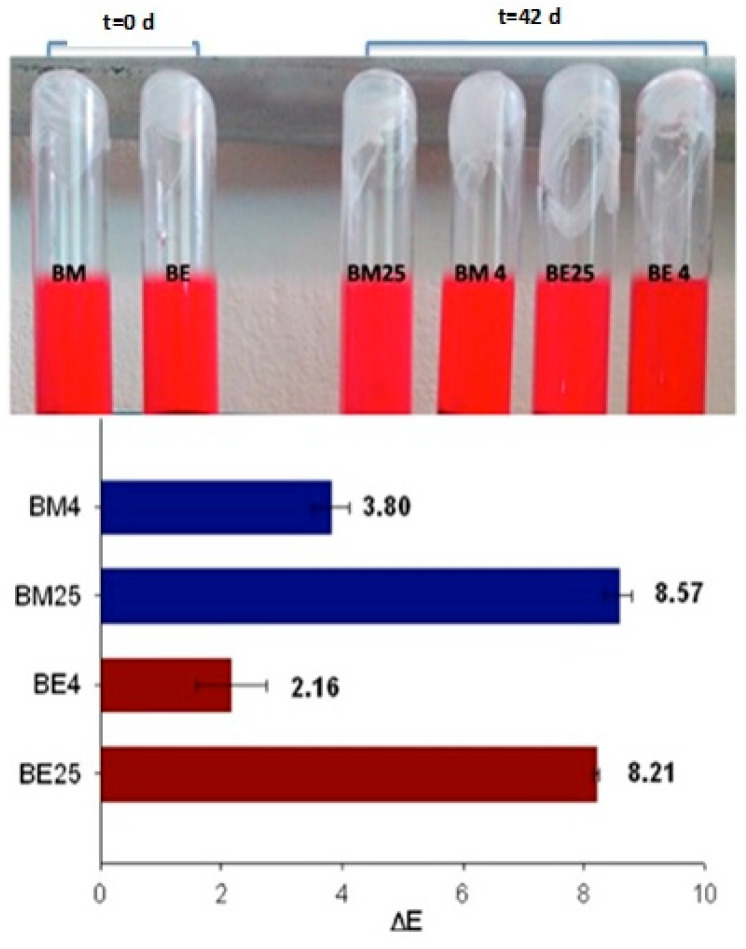
Appearance of the color of isotonic beverages at 0 and 42 days of storage (**top**) and the color differences (ΔE) in beverages after 42 days of storage under two temperatures (4 and 25 °C). BM4: beverage with AM stored at 4 °C; BM25: beverage with AM stored at 25 °C; BE4: beverage with AE at 4 °C; BE25; beverage with AE stored at 25 °C.

Differences between colors are described by the total difference between two colors in the three dimensions of the CIELab color space [23]. In the evaluated beverages, the color difference was primarily due to the significant effect (*p* < 0.0001) of temperature. In this regard, the beverages stored at 25 °C displayed higher color changes than those stored at 4 °C (Figure 2, top). At the end of the storage period, the beverages stored at 4 °C exhibited ΔE values of 3.8 in BM and 2.16 in BE. In the beverages stored at 25 °C, the ΔE values were considerably higher, but with no differences between BM and BE (Figure 2, bottom). A color difference of 0 to 1.5 can be considered small and imperceptible to visual observation; in the 1.5 to 5 intervals, the color difference can be distinguished, while the color difference can be evident for ΔE higher than 5 [23].

### 3.2. Total Soluble Solids (°Bx) and pH of Beverages

The TSS (in °Bx) of the beverage colored with AM were higher than those of the beverage colored with AE, a result that was possibly related to the partial solubilization of microcapsules from the moment they were added to the beverage. Tupuna–Yerovi et al. [24] reported similar results for a tangerine isotonic beverage colored with norbixin carotenoid added in microcapsules and free. In this case, the wall material of the microcapsules was gum Arabic that solubilized in the aqueous beverage and increase the TSS.

**Figure 3:**
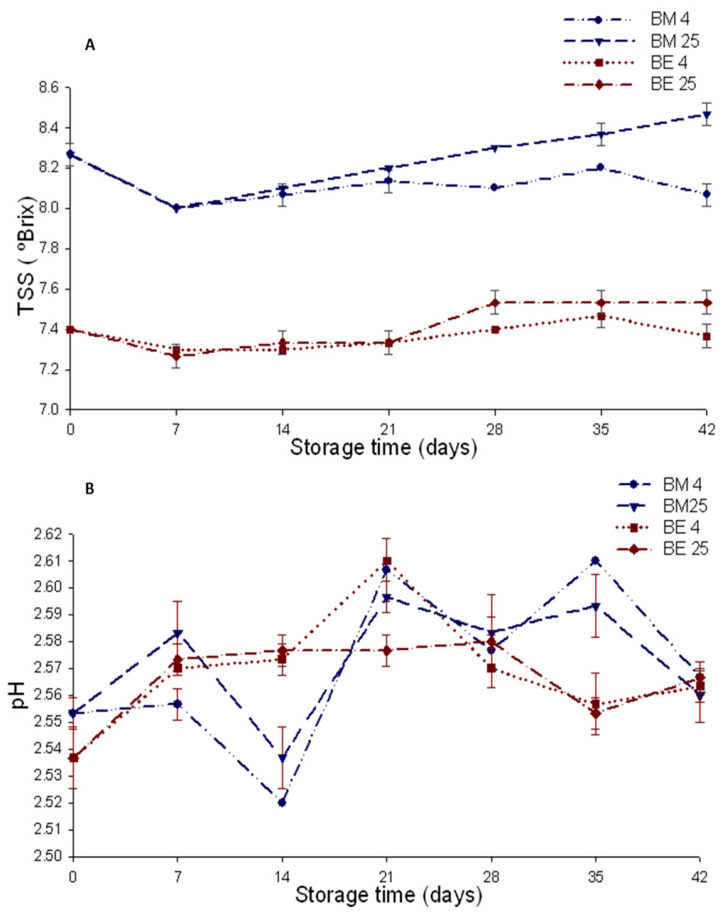
Total soluble solids (TSS) (**A**) and pH (**B**) in isotonic beverages with microencapsulated anthocyanins (BM4 and BM25) and anthocyanins in extract (BE4 and BE25) stored for 42 days.

The TSS increased in both beverages after 14 days of storage (dda), particularly in the beverages stored at 25 °C (Figure 3A). It is possible that increased TSS was produced by the hydrolysis of starches of the wall material of the microcapsules to sugars by the very acidic pH of the system and the storage temperature. Girones Villaplana et al. [16] reported an increase in soluble solids in isotonic drinks with added lyophilized berries stored for 70 days at 25 °C, which they related to the hydrolysis of starch in the berries into sugars. It is also possible that the increase in TSS was caused by the de-glycosylation of anthocyanins, which is the initial step in the thermal degradation process of these compounds [24].

The pH of the beverages varied between 2.53 and 2.55 and was maintained without significant changes during the storage period. At the end of storage, this variable exhibited values of 2.57 and 2.56 for the BM4 and BM25 beverages, respectively (Figure 3B). There was no significant difference for this variable between the beverages at the start and at the end of storage. The acid pH is a factor that favored anthocyanins stability in aqueous system [25]. In blueberry juice, the reduction of pH from 2.9, that is the original pH of the juice, to 2.1 before the pasteurization process reduce anthocyanins lost in about 6%. The juice at pH 2.1 retained 78% of anthocyanins vs. the control a pH of 2.9 that retained only 62%, what showed that during blueberry juice storage, the pH plays a role most relevant than temperature in anthocyanins retention [26].

### 3.3. Stability of Anthocyanins during Beverages Storage

**Figure 4:**
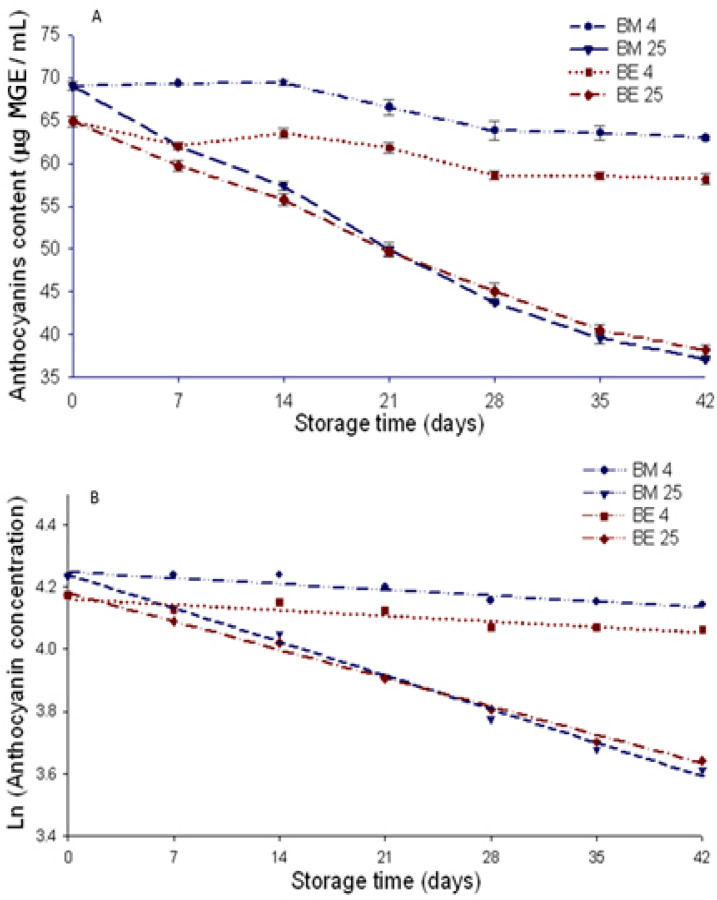
Anthocyanin content (**A**) and degradation kinetics (**B**) of chagalapoli anthocyanins added to an isotonic beverage in the form of microcapsules and as extract stored under two temperature conditions (4 and 25 °C). BM4: beverage with microcapsules stored at 4 °C; BM25: beverage with microcapsules stored at 25 °C; BE4: beverage with extract at 4 °C; BE25: beverage with extract at 25 °C.

The TAC of beverages at the beginning of storage was 64.9 and 69.04 µg MGE/mL for BM and BE, respectively. The TAC in the beverages decreased during storage, with the highest losses in beverages stored at 25 °C (Figure 4A). The degradation pattern of the anthocyanins in the beverages fit the first-order kinetics model defined by a straight line of negative slope obtained by graphing the natural logarithm of the concentration of anthocyanins vs. storage time (Figure 4B). This result coincides with reports of other authors in relation to anthocyanins in aqueous medium following first-order degradation kinetics [14,27]. The type of anthocyanins, being acylated or glycosylated seems to affect the degradation kinetics pattern [28]. The degradation kinetic of anthocyanins from purple potato (mainly acylated) fits better to a model of zero order [27]. The anthocyanins from chagalapoli are of glycosylated type [13].

The data showed a good fit to the first-order kinetics model (0.8424 < R2 < 0.9926), particularly those obtained in the beverage stored at 25 °C (Table 1). The reaction rate (*k*), half-life time (t_1/2_), and anthocyanin retention (AR) were affected by storage temperature but not by the form of anthocyanins added to the beverage (AM or AE). Beverages stored at 25 °C with AM (BM25) and AE (BE25) exhibited reaction rates of 0.00069 and 0.00053, respectively, which were not significantly different (*p* ≥ 0.05). In beverages stored at 4 °C, the reaction rate was significantly lower (*p* ≤ 0.05), without differences between beverages with AM (BM4) or AE (BE4) (Table 1).

González de Mejía et al. [22] found reaction rates of 0.09, 0.14, and 0.21 h for temperatures of 70, 80, and 90 °C, respectively, applied for five hours to an aqueous Kool-Aid^®^-based beverage colored with a semi-purified extract of purple corn anthocyanins. In another study, Aguilera et al. [14] reported *k* values of 0.0218 and 0.2134 weeks for a sports beverage storage at 4 and 25 °C, in this order, added with free semipurified anthocyanins from black bean coats. In both studies is evident that as high the temperature is as high as the *k* values. A higher *k* value indicates a faster rate of anthocyanins degradation.

Anthocyanins in beverages stored at 4 °C exhibited half-life values of 6019.5 h, without differences between the beverages with microencapsulated anthocyanins (BM4) and those with anthocyanin extract (BE4). In the beverages stored at 25 °C, the half-life of the anthocyanins was shorter, with values of 1337.7 and 1003.3 h in beverages BE25 and BM25, respectively.

The AR in beverages storage at 4 °C was higher with respect to that storage at 25 °C. AR was not significantly different (*p* ≥ 0.05) between the BM4 and BE4 beverages. In the beverages stored at 25 °C, BE25 displayed a higher AR (*p* ≤ 0.05) than that of BM25. As has been reported by other authors, low temperatures preserve well the anthocyanins in beverages [14,29]. Even if they are added as AM or AE. However, at room temperature storage (25 °C), chagalapoli AM added to the beverage degraded faster than AE. The higher susceptibility of AM to degrade compared with AE in the beverage stored at 25 °C might originate in its exposure to high temperature (160 ± 1 °C) during the spray-drying process used to obtain microcapsules, a condition to which the AE was not subjected. The maltodextrin: the capsule mixture used as wall material for the microencapsulation of the chagalapoli anthocyanins did not protect them in the aqueous media because the wall was probably solubilized. Anthocyanins degrade more rapidly when they are in aqueous media than in foods and are more unstable after being subjected to high temperatures [30]. Using other wall materials such as chitosan could improve the anthocyanin’s stability in an aqueous system particularly if they are added as nanoparticles, for not altering the beverage color due to their partial solubility [28].

The evaluated beverages had a pH of 2.56 to 2.57, which did not change significantly throughout storage (Figure 2B), which probably favored the anthocyanin’s stability. Under these pH conditions, it is possible that the predominant chemical form of chagalapoli anthocyanins was the flavylium cation [31], which is one of the more stable forms [10]. At very acidic pH (1–2), it has been demonstrated that monoglucoside anthocyanins, which are dominant in chagalapoli, are more stable than di-glucoside anthocyanins [32]. In an aqueous environment, degradation initiates with the hydrolysis of the glucosidic bond to produce monosaccharides and aglycones [25]. In the second step, excision of the aglycone occurs, which separates into a phenolic acid and phenolic aldehyde (the final products of degradation) [33].

### 3.4. HPLC Analysis of Anthocyanins in the Beverages

**Figure 5:**
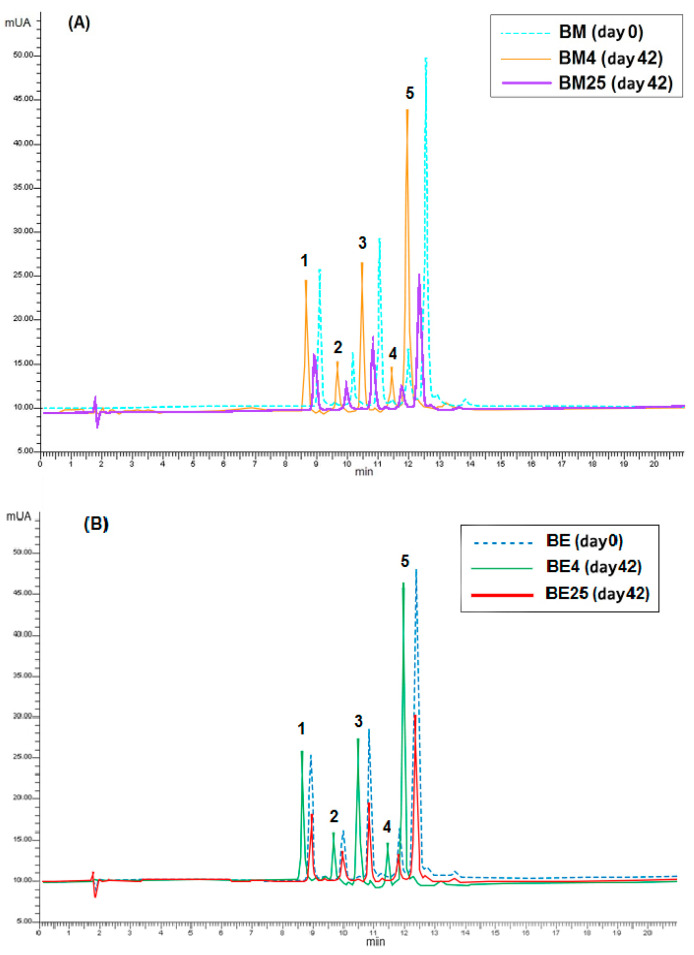
Anthocyanin profile of the isotonic beverage added with microcapsules (BM) (**A**) or extract (BE) (**B**), and stored at two temperatures (4 and 25 °C). Identity of peaks: (1) delphinidin 3-O-galactoside, (2) cyanidin 3-O-galactoside, (3) petunidin 3-O-galactoside, (4) peonidin 3-O-galactoside, and (5) malvidin 3-O-galactoside.

The chromatographic profile of chagalapoli anthocyanins consists of 12 anthocyanins, of which 10 have been identified. The five main anthocyanins of this fruit were as follows: malvidin 3-O-galactoside (mv 3-O-gal), petunidin 3-O-galactoside (pt 3-O-gal), delphinidin 3-O-galactoside (dp 3-O-gal), cyanidin 3-O-galactoside (cy 3-O-gal), and peonidin 3-O-galactoside (pn 3-O-gal) [13]. These anthocyanins correspond to peaks 1 to 5 in Figure 5, which illustrates the degradation of chagalapoli anthocyanins in the beverage to which they were added as AM (Figure 5A) or as AE (Figure 5B), and stored at 4 and 25 °C.

The five anthocyanins that were monitored in the beverages during storage represent more than 90% of the total anthocyanins in chagalapoli [13]. Differences in total anthocyanins measured by HPLC in the beverages at time 0 with respect to those obtained by the spectrophotometric method are due to the contribution of minor anthocyanins that were not quantified by HPLC.

In the beverages stored at 4 °C, the initial concentration of anthocyanins was maintained without changes at the end of storage, regardless of the form in which they were added to the beverage (AM or AE). In the beverage with AE, the concentration at the end of storage was slightly higher than that at the beginning because the anthocyanins dp 3-O gal, cy 3-O gal, and pt 3-O gal had a higher concentration at the end of storage under this temperature (Table 2). In the beverages stored at 25 °C, there was a 59.13% total loss of anthocyanins in BM and a 46.58% loss in BE beverages. Under this storage condition, all anthocyanins reduced their presence.

Regarding the mono glycosides of anthocyanin in the beverages, the most stable at 25 °C was cyanidin 3-O-galactoside, added either as microcapsules or as extract. Peonidin 3-O-galactoside was the most susceptible to degrade when was added as microcapsules, while delphinidin 3-O-galactoside was the most unstable when added as an extract. Anthocyanin type affects stability. Hellstrom et al. [34] found greater stability of cyanidin 3-glucoside compared with delphinidin 3-glucoside in currant juice stored at 21 °C. Kurtulbas et al. [35] reached the same results in relation to the high stability of cyanidin 3- glucoside during the storage of sour cherry extract at 25 °C. Besides, under neutral pH solutions, red cabbage mono-acylated anthocyanins bleached before acylated anthocyanins [36]. The high stability of acylated anthocyanins is associated with the complex structure that allowed them to be protected from the nucleophilic attack of ions to the chromophore site [37].

## 4. Conclusions

The stability of microencapsulated anthocyanins was equal (*p* ≥ 0.05) to that of extract or free anthocyanins in the isotonic beverage stored at 4 °C. In the beverage stored at 25 °C, microencapsulated anthocyanins exhibited lower (*p* ≤ 0.05) stability than free anthocyanins based on the k, t_1/2,_ and AR variables. Under two storage temperatures, the degradation of anthocyanins was fit to a first-order kinetics model. Of the chagalapoli anthocyanins, cyanidin 3-O-galactoside was the most stable in the isotonic beverage, regardless of whether it was added in the microencapsulated form or free. Chagalapoli anthocyanins, both as microcapsules or as an extract, are an option for adding natural color to isotonic beverages. Future studies can consider using nanocapsules with wall materials less soluble in an aqueous system to evaluate the anthocyanin’s stability and the impact on the color of the products.

## Figures and Tables

**Table 1:** Kinetic degradation parameters of chagalapoli anthocyanins added to an isotonic beverage as microcapsules and in extract and stored at 4 and 25 °C in the absence of light.

Treatments	R^2^	*k* × 10^−3^ (h^−1^)	t_1/2_ (h)	AR (%)
BM4	0.8508 ^b^	0.11 ^b^	6019.5 ^a^	91.21 ± 0.28 ^a^
BM25	0.9917 ^a^	0.69 ^a^	1003.3 ^b^	53.72 ± 0.27 ^c^
BE4	0.8424 ^b^	0.11 ^b^	6019.5 ^a^	89.63 ± 0.22 ^a^
BE25	0.9926 ^a^	0.53 ^a^	1337.7 ^b^	58.83 ± 1.37 ^b^

The values presented are the mean of three replicates. *k*: reaction rate constant, t_1/2:_ half-life time of anthocyanins, AR: anthocyanin retention. The same letters within the same column indicate no significant difference (α = 0.05). BM4: beverage with microcapsules (4 °C); BM25: beverage with microcapsules (25 °C); BE4: beverage with extract (4 °C); BE25: beverage with extract (25 °C); AR: anthocyanin retention.

**Table 2:** Quantities of the five main anthocyanins of chagalapoli in beverages were analyzed by HPLC at the beginning and end of the storage period at two temperatures.

Beverage	Time	Dp 3-O*-*Gal	Cy 3-O-Gal	Pt 3-O-Gal	Pn 3-O-Gal	Mv 3-O-Gal	Total ACNs
	(days)	(µg/mL)	(µg/mL)	(µg/mL)	(µg/mL)	(µg/mL)	(µg/mL)
BM	0	10.26 ± 0.09 ^b^	4.43 ± 0.11 ^a^	13.07 ± 0.18 ^a^	4.95 ± 0.04 ^a^	30.82 ± 0.02 ^a^	63.53 ± 0.04 ^a^
BM4	42	10.95 ± 0.16 ^a^	4.30 ± 0.10 ^a^	12.95 ± 0.12 ^a^	4.66 ± 0.25 ^a^	30.17 ± 0.54 ^ab^	63.03 ± 0.10 ^a^
BM25	42	4.36 ± 0.15 ^d^ (42.49)	1.99 ± 0.12 ^b^ (44.92)	5.34 ± 0.22 ^c^ (40.85)	1.92 ± 0.45 ^b^ (38.78)	12.36 ± 0.74 ^d^ (40.10)	25.97 ± 0.11 ^e^ (40.87)
BE	0	9.98 ± 0.03 ^b^	4.17 ± 0.13 ^a^	12.49 ± 0.13 ^a^	4.57 ± 0.14 ^a^	29.47 ± 0.35 ^ab^	60.68 ± 0.17 ^c^
BE4	42	10.83 ± 0.06 ^a^	5.02 ± 1.00 ^a^	13.00 ± 0.44 ^a^	4.21 ± 0.57 ^a^	29.07 ± 0.41 ^b^	62.13 ± 0.36 ^b^
BE25	42	5.21 ± 0.10 ^c^ (52.20)	2.49 ± 0.09 ^b^ (59.71)	6.59 ± 0.18 ^b^ (52.76)	2.49 ± 0.19 ^b^ (54.48)	15.64 ± 0.18 ^c^ (53.07)	32.42 ± 0.20 ^d^ (53.42)

ACNs: anthocyanins; BM: beverage with microcapsules; BM4: beverage with microcapsules at 4 °C; BM25: beverage with microcapsules at 25 °C; BE: beverage with extract; BE4: beverage with extract at 4 °C; BE25: beverage with extract at 25 °C. Values are the mean of three replicates ± standard deviation. The same letter within a column indicates no statistical difference (α = 0.05). Values in parentheses represent the retention percentage of anthocyanins at 42 days of storage. Those values with a + means that anthocyanin increase during storage.

## Data Availability

The data presented in this study are available on request from the corresponding author.

## Supplementary Materials

The following supporting information can be downloaded at: https://www.mdpi.com/article/10.3390/foods12102009/s1, Table S1: Correlation coeficients between the Luminosity (L) and the anthocyanins content of the beverages at the different storage temperatures (4 and 25 °C).
