# Supplementary material for: Color and Stability of Anthocyanins of Chagalapoli (Ardisia compressa K.) Fruit Added to an Isotonic Beverage as Microcapsules and as Free Extract
Source: Foods. 2023 May 16;12(10):2009. doi: 10.3390/foods12102009 (PMC10217480; doi:10.3390/foods12102009)
Supplement: Supplementary file 1 [file foods-12-02009-s001.zip › foods-2336531-supplementary.pdf]

Table S1. Correlation coeficients between the Luminosity (L) and the anthocyanins content of the beverages at the different storage temperatures (04 and 25 °C).

	BM25	BM04	BE25	BE04
LM25	−0.9698			
LM04		−0.8851		
LE25			−0.9477	
LE04				−0.8501

BM25: beverage added with anthocyanin microcapsules, storage at 25 °C, BM04: beverage added with anthocyanin microcapsules storaged at 04 °C, BE: beverage added with anthocyanin extract storage at 25 °C, BE: beverage added with anthocyanin extract storaged at 04 °C.
